# Sudden Death in a Dentist’s Office

**Published:** 1867-03

**Authors:** 


### *Sudden Death in a Dentist's Office.*

Last week Edmund Kerosin, a young man 23 years old, entered the office of Dr. Ralph Lee, a dentist of this city, to have a tooth extracted. Anæsthesia was produced by nitrous oxyd gas, a cork having been placed between the teeth to keep the mouth open. As the tooth was extracted, we understand, it slipped from the forceps, and, with the cork, was drawn into the mouth. The tooth was subsequently thrown from the stomach, but the cork—which does not seem to have been missed—entered the larynx, and by its presence there caused suffocation and death in an hour. A post mortem revealed the presence of the cork in the larynx, and the cause of death. This case and its lamentable result should serve as a caution to those who employ such adjuncts in the dental laboratory, and the physician who may be suddenly summoned to patients in a dentist's office, should bear in mind the possibility of an accident like this, and be prepared to open the larynx, if need be, which, in this instance, would, in all probability, have given instant relief, and saved the life of the young man.—*Phil. Med. and Surg. Reporter.*

